# Molecular Identification of *Echinococcus multilocularis* Infection in Small Mammals from Northeast, Iran

**DOI:** 10.1371/journal.pntd.0002313

**Published:** 2013-07-11

**Authors:** Molouk Beiromvand, Lame Akhlaghi, Seyed Hossein Fattahi Massom, Ahmad Reza Meamar, Jamshid Darvish, Elham Razmjou

**Affiliations:** 1 Department of Parasitology and Mycology, School of Medicine, Tehran University of Medical Sciences, Tehran, Iran; 2 Department of Parasitology, School of Medicine, Ahvaz Jundishapur University of Medical Sciences, Ahvaz, Iran; 3 Department of Thoracic Surgery, Ghaem Educational, Research and Treatment Center, Mashhad University of Medical Sciences, Mashhad, Iran; 4 Department of Rodents Research, Ferdowsi University of Mashhad, Mashhad, Iran; Queensland Institute of Medical Research, Australia

## Abstract

**Background:**

Alveolar echinococcosis is a zoonotic disease caused by the metacestode of *Echinococcus multilocularis*. Many species of small mammals, including arvicolid rodents or *Ochotona* spp., are natural intermediate hosts of the cestode. The main aim of this study was to identify natural intermediate hosts of *E. multilocularis* in Chenaran County, Razavi Khorasan Province, northeastern Iran, where the prevalence of infected wild and domestic carnivores is high.

**Methodology/Principal Findings:**

A program of trapping was carried out in five villages in which this cestode was reported in carnivores. The livers of 85 small mammals were investigated for the presence of *E. multilocularis* infection using multiplex PCR of mitochondrial genes. Infections were identified in 30 specimens: 23 *Microtus transcaspicus*, three *Ochotona rufescens*, two *Mus musculus*, one *Crocidura gmelini*, and one *Apodemus witherbyi*.

**Conclusions/Significance:**

A range of small mammals therefore act as natural intermediate hosts for the transmission of *E. multilocularis* in Chenaran County, and the prevalence suggested that *E. multilocularis* infection is endemic in this region. The existence of the life cycle of this potentially lethal cestode in the vicinity of human habitats provides a significant risk of human infection.

## Author Summary

Small mammals, especially rodents, coexist with humans, particularly in rural areas where they become infected with the larval stages of *Echinococcus multilocularis* via ingestion of eggs in feces from infected carnivores. As prey for carnivores, small mammals have an important role in the life cycle of *E. multilocularis*, the agent of a serious zoonotic disease, alveolar echinococcosis, infecting people in most northern hemisphere countries. We detected *E. multilocularis* infection in the liver of small mammals in villages of the Chenaran region of northeastern Iran, where we previously found adult *E. multilocularis* and/or eggs in all wild, and some domestic, carnivores examined. Several species of small mammals, especially the Transcaspian vole (*Microtus transcaspicus*), were natural hosts of this cestode. Since infected voles are a good marker of soil contamination with *E. multilocularis* eggs, they are an indicator of the infection risk for inhabitants. There is a need to educate local populations about risk of infection to avoid transmission of this pathogenic parasite.

## Introduction

Echinococcosis is a near cosmopolitan parasitic disease caused by the cestode *Echinococcus* [1], [2], [3]. The potentially fatal zoonotic disease, alveolar echinococcosis, is caused by the metacestode of *E. multilocularis*, which has a sylvatic cycle, comprising wild carnivores as definitive hosts and more than 40 species of small mammals, including arvicolid rodents and the lagomorph *Ochotona* spp., as intermediate hosts [2], [4], [5]. Humans are accidental and aberrant intermediate hosts infected by parasite eggs ingested in contaminated food or by direct contact with infected definitive hosts [5], [6].

*Echinococcus multilocularis* distribution is restricted to the northern hemisphere, including Central Europe, the Near East, Russia, Central Asian republics, northern Japan, parts of North America [7], [8], [9], [10], [11], and some countries of the Middle East [12], [13]. In Iran, information about *E. multilocularis* infection is limited to a few studies restricted to the northwestern areas of the country [14], [15], [16], [17]. The first study in Iran, conducted in 1971 on the Moghan Plain, reported *E. multilocularis* infection in 10% of red foxes (*Vulpes vulpes*) [14], [15], although its metacestode was not found in any of the 5000 rodents examined (unpublished data). In 1992, a further study of 130 wild carnivores and 1500 rodents showed 22.9% of red foxes and 16% of jackals (*Canis aureus*) infected with adult *E. multilocularis* but no metacestodes in the rodents [12], [17].

Investigation of definitive and intermediate hosts of *E. multilocularis* in other parts of the country has been neglected. Recently, following a few reported cases of human alveolar echinococcosis [18] (E. Razmjou, unpublished data), a morphological and molecular survey was carried out on wild and domestic carnivores from the Chenaran area in northeastern Iran [19]. Based on this study, the high prevalence of carnivores infected with *E. multilocularis* indicates that the life cycle of *E. multilocularis* is being maintained here, and Razavi Khorasan Province was shown to be an endemic area [19]. Therefore, the role of dogs, foxes, jackals, wolves, and hyenas was confirmed as a definitive host. However, no data were available on the intermediate hosts of *E. multilocularis* in the Chenaran area.

To determine the *E. multilocularis* life cycle in a specific region, study of its potential intermediate hosts is imperative, since voles have a small home-range and infected voles are a good marker for the presence of *E. multilocularis* eggs [20], thus indicating the risk of human infection at a local level [21]. This investigation was carried out to identify the natural intermediate hosts and determine the prevalence of infection in Chenaran County to confirm the life cycle of this pathogenic cestode in this region.

## Materials and Methods

### Ethics statement

For investigating the presence of *E. multilocularis* infection, small mammals were trapped under license from the Iran Environment Protection Organization. Animals were handled according to the American Society of Mammalogists (ASM) guidelines for animal research, and the experimental protocols were reviewed and approved by the Ethics Committee of Tehran University of Medical Sciences (Approval No 759-2008). The inhabitants of Chenaran County villages, on whose land the specimens were collected, gave their informed consent for the trapping.

### Study area

**Figure 1:**
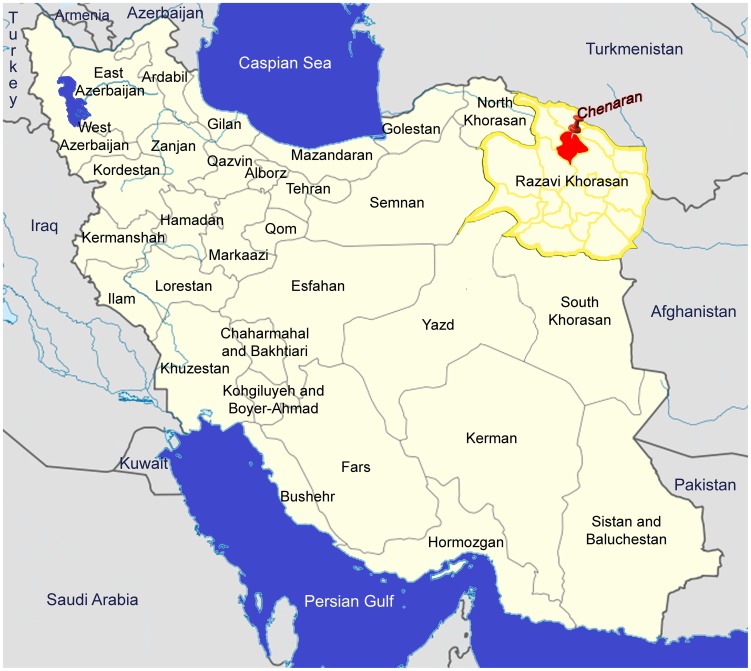
Map of Iran. Razavi Khorasan Province is highlighted by yellow border. The study area, Chenaran County, is indicated by red (red pin). Based on the map of Iran at [19].

Razavi Khorasan Province is located in northeastern Iran in the vicinity of Turkmenistan and Afghanistan (Figure 1). Chenaran (36°38′N, 59°7′E), one of 19 counties in the province, is northwest of the capital, Mashhad, and had a population of approximately126,000 in 2011 [22]. It is a region of highlands, located between Binalood Heights and the Hezar Masjed Mountains at an elevation of 1400–1600 m. Average temperature in winter is 4.1°C; colder at higher elevations. Mean summer temperature is 23.9°C. Annual precipitation averages 212.6 mm with the lowest rainfall in summer. Most villages of Chenaran County are located in valleys with natural rivers as a source of water for fruit gardens and household use. The grasslands and high soil moisture in these areas provides suitable habitat for small mammals that attract predators, and likely good conditions for taeniid egg survival [23].

### Sampling

Trapping was conducted in October 2010 and July 2011 in five villages that reported high rates of *E. multilocularis* in carnivores [19]. Thirty small mammals were collected specifically for this investigation, and 55 others were trapped by inhabitants of villages to reduce rodent damage to trees and gardens. Small Sherman live traps (25×10×10 cm) were baited with cheese, muffins spread with butter, walnuts, or fruit. Trapping sites included gardens, river banks, storage rooms, and areas near burrows. All traps were checked twice daily and trapped animals were collected, labeled with date and place of sampling, and stored at −20°C.

Small mammals were identified using standard morphological criteria [24]. They were dissected, and the thoracic and peritoneal cavity and visceral organs, particularly the liver, were examined macroscopically for cysts of *E. multilocularis* and other parasites. Distinguishable lesions and the liver of all specimens were excised and preserved in 80% ethanol for molecular examination.

### DNA extraction

For molecular analysis, the ethanol was discarded and liver samples were hydrated with 0.9% NaCl overnight. The liver was forced through a 420 µm mesh sieve and washed with PBS buffer. The liver puree and PBS buffer were transferred to a 15 ml falcon tube, centrifuged at 800×g for 10 min, and 400 µl of the cell suspension in PBS buffer, equal to approximately 25 mg liver tissue, was transferred to 2 ml tubes. DNA was extracted using the QIAamp DNA Mini kit (QIAGEN, Germany) tissue protocol, according to manufacturer's instructions, and the Verweij et al. [25] protocol with slight modification as described [19]. DNA was stored at −20°C until molecular analysis.

### Multiplex PCR

All DNA samples were amplified using primer pairs, and conditions in multiplex PCR as described for detection of *E. multilocularis*, *E. granulosus*, and *Taenia* spp. infections [26]. The primer pairs were arranged to amplify partial sequences of the mitochondrial genes for NADH dehydrogenase subunit 1 (*nad1*) for detection of *E. multilocularis*, and the small subunit of ribosomal RNA (*rrnS*) for detection of *E. granulosus* and *Taenia* spp.

Multiplex PCR was conducted on a final volume of 25 µl reaction mixture according to conditions and parameters previously described [19]. Amplification products were visualized by 2% (W/V) agarose gel electrophoresis, and the 395, 117, and 267 bp expected fragments were examined for presence of *E. multilocularis*, *E. granulosus*, and *Taenia* spp., respectively. In all PCR reactions, distilled water was used as a negative control and standard DNA of *E. multilocularis*, *E. granulosus*, and *Taenia hydatigena* (provided by Professor Deplazes, Institute of Parasitology, Zurich, Switzerland) as positive controls, to validate the PCR reaction results. In order to decrease inhibition factors and increase likelihood of detecting positive samples, we diluted DNA samples with distilled water and conducted multiplex PCR on serial dilutions of DNA. The optimal volume of DNA for PCR reaction was in the range 0.25–2 µl in 25 µl of reaction mixture.

For further confirmation, samples were examined using single PCR with primers Cest1/Cest2 [26] and EM-H15/EM-H17 [20] for *E. multilocularis*, Cest4/Cest5 for *E. granulosus*, and Cest3/Cest5 for *Taenia* spp. [26] and sequencing of *E. multilocularis* positive samples. *Echinococcus multilocularis* amplified fragments were extracted from agarose gels using the QIAquick Gel Extraction Kit (QIAGEN, Germany), according to the manufacturer's instructions and were sequenced on both strands with primers Cest1/Cest2 (Bioneer, Korea). The sequence results were compared with the Genbank database using the DNASIS MAX (version 2.09; Hitachi, Yokohama, Japan) software.

## Results

### Identification of small mammals

Base on morphological criteria, the 85 small mammals trapped in five villages of Chenaran County were classified into six species of the four families Cricetidae, Muridae, Soricidae, Ochotonidae. The majority of small mammals caught were *Microtus transcaspicus* (63.5%, 54/85) of the Cricetidae. Muridae was second, with 15 *Mus musculus* (17.6%), nine *Apodemus witherbyi* (10.6%), and one *Nesokia indica* (1.2%). Four were *Ochotona rufescens* (4.7%), and two were *Crocidura gmelini* (2.4%) of the non-rodent families Ochotonidae and Soricidae (Table 1).

**Table 1:** Prevalence of *E. multilocularis* in small mammal species.

Host species	*M. transcaspicus*	*M. musculus*	*A. witherbyi*	*O. rufescens*	*C. gmelini*	*N. indica*	Total
Village (altitude m)	aN_P_/N_C_ (%)^b^	N_P_/N_C_ (%)	N_P_/N_C_ (%)	N_P_/N_C_ (%)	N_P_/N_C_ (%)	N_P_/N_C_ (%)	N_P_/N_C_ (%)
cV1 (1558)	18/30 (60)	1/7 (14.3)	1/6 (16.7)	3/4 (75)	1/1 (100)	0/1 (0)	24/49 (49.0)
V2 (1523)	0/9 (0)	0/0	0/2 (0)	0/0	0/0	0/0	0/11 (0)
V3 (1428)	2/12 (16.7)	0/0	0/1 (0)	0/0	0/0	0/0	2/13 (15.4)
V4 (1452)	3/3 (100)	0/0	0/0	0/0	0/1 (0)	0/0	3/4 (75)
V5 (1469)	0/0	1/8 (12.5)	0/0	0/0	0/0	0/0	1/8 (12.5)
Total	23/54 (42.6)	2/15 (13.3)	1/9 (11.1)	3/4 (75)	1/2 (50)	0/1 (0)	30/85 (35.3)

a N_C_: Number of captured small mammals, N_P_: Number of *E. multilocularis* positive small mammals.

b percent infection,

c V1–V5: village1–5.

### Morphological examination

Macroscopic examination of visceral organs showed liver cysts in nine of 85 (10.6%) animals. Liver cysts were isolated from six of 54 (11.1%) *M. transcaspicus* and two of 15 (13.3%) *M. musculus*. Three cysts were observed, in liver of one *C. gmelini*.

### Molecular analysis

**Figure 2:**
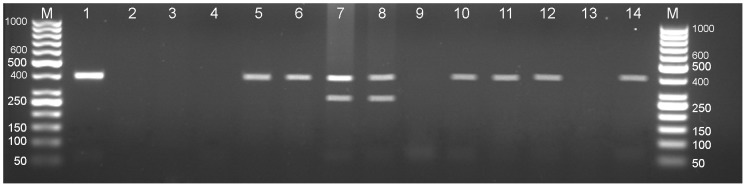
Multiplex PCR amplification of mitochondrial genes of *E. multilocularis* and *Taenia* spp. of DNA extracted from liver of small mammals. Lane M, 50 bp DNA ladder (Fermentas; Cat No SM0373); Lane 1, positive control, a standard DNA of *E. multilocularis* (395 bp); Lane 2, negative control; Lane 5, 6, 10–12 *E. multilocularis*; Lane 7 and 8, mixed infection of *E. multilocularis* (395 bp) and *Taenia* spp. (267 bp); Lane 3, 4, 9, and 13 negative samples.

Multiplex PCR showed 30 of 85 captured specimens (35.3%) to be infected with *E. multilocularis* and 14 (16.5%) infected with *Taenia* spp. by amplification of 395 bp fragment of *nad1* and 267 bp fragment of *rrnS*, respectively (Figure 2).

*Echinococcus multilocularis* infection was identified in liver of 23 of 54 *M. transcaspicus* (42.6%), three *O. rufescens* (75.0%; 3/4), two *M. musculus* (13.3%; 2/15), one *C. gmelini* (50.0%; 1/2), and one *A. witherbyi* (11.1%; 1/9). *Taenia* spp. were found in liver of nine *M. transcaspicus* (16.7%; 9/54), two *M. musculus* (13.3%; 2/15), one *C. gmelini* (50.0%; 1/2), and one *A. witherbyi* (11.1%; 1/9). The only *N. indica* specimen captured was infected with *Taenia* spp. The single PCR amplifications confirmed the results of multiplex PCR.

*Echinococcus multilocularis* and *Taenia* spp. co-infections were revealed in 11 of 33 PCR positive samples by amplification of two species-specific fragments (Table 2). A single amplicon detected 19 *E. multilocularis* (22.4%) and three *Taenia* spp. (3.5%) infected small mammals (Table 2).

**Table 2:** Number (%) of single and co-infections with *E. multilocularis* and *Taenia* spp. in liver samples of small mammals.

Host species	N	*E. multilocularis*	*Taenia* spp.	*E. multilocularis+Taenia* spp.
*M. transcaspicus*	54	16 (29.6%)	2 (3.7%)	7 (13.0%)
*M. musculus*	15	0	0	2 (13.3%)
*A. witherbyi*	9	0	0	1 (11.1%)
*O. rufescens*	4	3 (75.0%)	0	0
*C. gmelini*	2	0	0	1 (50.0%)
*N. indica*	1	0	1 (100.0%)	0
Total	85	19 (22.4%)	3 (3.5%)	11 (12.9%)

N, sample size.

Fifty-two liver samples were negative for *E. multilocularis* and *Taenia* spp. by all methods used. *Echinococcus granulosus* infection was not found in any liver sample.

All positive samples were confirmed as *E. multilocularis* using sequencing of the *nad1* gene. The alignment of amplified *nad1* sequences showed 100% identity with published reference sequences for *E. multilocularis*. The nucleotide sequence of five *E. multilocularis* amplified *nad1* genes from five small mammal species were deposited in the DDBJ/EMBL/GenBank nucleotide sequence database under accession number AB720065–69.

## Discussion

Although previous studies of definitive hosts have revealed that northwestern Iran is an endemic focus for *E. multilocularis*, its metacestode stages have not been found [12], [17]. The most recent investigation, using morphological and molecular methods, indicated high endemicity in the newly surveyed Chenaran County, with 100% prevalence of infection in wild carnivores and 6.5% in domestic and stray dogs [19]. As the presence of both definitive and intermediate hosts is required for establishment and maintenance of the life cycle, finding a high level of *E. multilocularis* infection (35.3%) among 85 small mammals belonging to two rodent families, Cricetidae and Muridae, and two non-rodent families, Ochotonidae and Soricidae, has confirmed the existence of the *E. multilocularis* life cycle in Chenaran County.

Reports of the prevalence of *E. multilocularis* infection have ranged from less than 1% to more than 80% in small mammals of the Soricidae, Talpidae, Sciuridae, Cricetidae, Arvicolidae, Muridae, Dipodidae, and Ochotonidae [2], [12], [27], [28]. This wide variation might be due to a wide spectrum of sensitive intermediate hosts [2] as well as to the number of investigated hosts and the diagnostic methods used [12]. The rate of infection in our study (35.3%) was lower than that found in some regions [2], [12], [27], but higher than reported in others [29], [30], [31]. The differing findings might be the result of identification based on gross and microscopic appearance of lesions found by histology [32], [33] or conducting PCR only on visually unidentifiable lesions [29], [30], [31]. In our study, cysts were detected in only 10.6% of the 85 investigated liver samples by direct examination, but this increased to 38.8% positive *E. multilocularis* and *Taenia* spp. infection with multiplex PCR on liver of all sampled specimens. It may be assumed that this finding reflected the complexity of distinguishing small immature cysts [34], especially in animals less than three months old [29], or in atypical or calcified liver lesions less than 5 mm in diameter [27], [35] by microscopic examination, while 14 pg of DNA can be detected by multiplex PCR [36]. An experimental infection of *Microtus arvalis* showed that PCR gives the only definitive diagnosis in lesions of less than two-weeks duration [37]. This may be the consequence of protoscoleces in the metacestode of *E. multilocularis* development extending over the course of 2–4 months in the liver of its natural intermediate host [12]. Stieger et al. [20] showed that *E. multilocularis*-specific PCR of 161 morphologically unidentifiable liver lesions of *Arvicola amphibius* (formerly *A. terrestris*) found 55 (34.2%) positive for *E. multilocularis* infection, increasing the detected prevalence of *E. multilocularis* in *A. amphibius* from 2.9% (26/889) to 9.1% (81/889) [20]. In a study in Geneva, Switzerland, in which 658 non-commensal rodents were investigated using morphological and molecular methods, metacestodes of *E. multilocularis* were detected in 2 adult *A. amphibius*, while PCR identified *E. multilocularis* infection in 29/79 *A. amphibius*, 3/4 *M. arvalis*, and 6/9 *Myodes glareolus* which was not found using morphological methods [21].

*Microtus transcaspicus* was the most frequently captured species (63.5%) and may be the dominant small mammal in the Chenaran area. It seems that this location, at an elevation of 1400–1600 m, having moist soil with trees and shrubs along river valleys is a suitable habitat for the Transcaspian vole (*M. transcaspicus*). Factors such as high elevation, low temperatures, high precipitation, moist soil, and an abundance of green vegetation provide suitable conditions for survival of *E. multilocularis* eggs [29], [30], [38] in feces of infected wild carnivores [19], in the studied habitats of small mammals. In central Europe, the main intermediate hosts are *M. arvalis* (common vole), *A. amphibius* (water vole), and *Ondatra zibethicus* (muskrat) [12], while in our study area, the higher density of *M. transcaspicus*, along with a high prevalence of infection, suggested an important role for this rodent in the *E. multilocularis* life cycle.

Although the previous study in this area showed high rates of *E. granulosus* infection in carnivores [19], no infection was identified in the small mammals examined. The first molecular identification of natural *E. granulosus* infection was reported in a single ground squirrel (*Spermophilus dauricus*), one of 500 small mammals trapped in northwest China, an endemic area for both *E. granulosus* and *E. multilocularis* [39]. While susceptible to *E. multilocularis*, *E. granulosus* infections have been seldom observed in rodents [40]. For detection of infected hosts, it may be necessary to investigate a greater number, and additional genera, of small mammals.

The presence of *Taenia* spp. in 14 of 85 (16.5%) specimens investigated, with 11 (12.6%) found co-infected with *E. multilocularis* by multiplex PCR, is a good indicator of contamination of the environment with taeniid eggs. Voles are natural intermediate hosts of several zoonotic helminthes, including *E. multilocularis*, *T. taeniaeformis*, *T. crassiceps*, and *Toxocara canis* that can infect humans who ingest eggs excreted by the final hosts [41]. Under suitable conditions, taeniid eggs might survive up to eight months and can be spread by shoes, animal paws, flies, or other vectors, infecting small mammals, humans, and other intermediate hosts in the endemic area [42].

In conclusion, the presence of infection in small mammals suggests the active transmission of *E. multilocularis* in the selected area. The existence of the life cycle of this potentially lethal cestode in the vicinity of human habitats provides a significant risk of human infection.

It is recommended that an extensive survey be conducted to investigate the prevalence of *E. multilocularis* in humans and domestic ungulates in Razavi Khorasan Province. In addition, there is a need to educate the local population about the infection, and programs for reducing the risk of transfer of infection to human and domestic animals should be initiated in Chenaran rural areas. As several human cases have been reported in other parts of Iran [43], further studies to investigate the life cycle of *E. multilocularis* in other parts of the country is recommended.
